# *Epichloë scottii* sp. nov*.*, a new endophyte isolated from *Melica uniflora* is the missing ancestor of *Epichloë disjuncta*

**DOI:** 10.1186/s43008-022-00088-0

**Published:** 2022-02-03

**Authors:** Torsten Thünen, Yvonne Becker, Murray P. Cox, Samad Ashrafi

**Affiliations:** 1 Institute for Crop and Soil Science, Julius Kühn Institute (JKI) – Federal Research Centre for Cultivated Plants, Bundesallee 58, 38116 Braunschweig, Germany; 2 Institute for Epidemiology and Pathogen Diagnostics, Julius Kühn Institute (JKI) – Federal Research Centre for Cultivated Plants, Messeweg 11/12, 38104 Braunschweig, Germany; 3 Statistics and Bioinformatics Group, School of Fundamental Sciences, Massey University, Palmerston North, New Zealand

**Keywords:** *Epichloë*, *Melica uniflora*, New species, Alkaloid profile, BUSCO multigene-phylogeny, Oxford nanopore, Telomere-to-telomere de novo genome assembly

## Abstract

Here we describe a new, haploid and stroma forming species within the genus *Epichloë*, as *Epichloë scottii* sp. nov. The fungus was isolated from *Melica uniflora* growing in Bad Harzburg, Germany. Phylogenetic reconstruction using a combined dataset of the *tubB* and *tefA* genes strongly support that *E. scottii* is a distinct species and the so far unknown ancestor species of the hybrid *E. disjuncta*. A distribution analysis showed a high infection rate in close vicinity of the initial sampling site and only two more spots with low infection rates. Genetic variations in key genes required for alkaloid production suggested that *E. scottii* sp. nov. might not be capable of producing any of the major alkaloids including ergot alkaloid, loline, indole-diterpene and peramine. All isolates and individuals found in the distribution analysis were identified as mating-type B explaining the lack of mature stromata during this study. We further release a telomere-to-telomere de novo assembly of all seven chromosomes and the mitogenome of *E. scottii* sp. nov.

## INTRODUCTION

*Epichloë* species (*Clavicipitaceae, Hypocreales*) live in a pleiotropic, constitutive symbiosis with grasses of the subfamily *Pooideae*. During the vegetative state, they colonize the intercellular space of the aerial plant tissue without causing any visible pathogenic symptoms (Scott et al. 2012). Most species are capable of vertical transmission by infection of ovules of developing seeds (Schardl et al. 2004). For species reproducing asexualy, distribution via clonal growth by tillering of the host and seed transmission are the only ways of reproduction. For sexual morphs of *Epichloë*, horizontal transmission starts with a switch from restricted endobiotic to proliferative epibiotic growth. This results in the forming of stromata, which enclose the developing inflorescences causing the typical “choke disease” (Scott and Schardl 1993). The mating system of *Epichloë* is bipolar and heterothallic. Flies act as the vector and transfer spermatia to stromata of the opposite mating type. After karyogamy and meiosis, ascospores are ejected and infect new host plants.

Most *Epichloë* species known only as an a asexual morph arose from interspecific hybridizations of different haploid *Epichloë* ancestors (Schardl 2010). The genus *Epichloë* currently comprises 15 haploid and 24 hybrid species (Leuchtmann et al. 2014; Leuchtmann et al. 2019; Campbell et al. 2017; Shymanovich et al. 2017; Tian et al. 2020) with *E. sinensis*, a hybrid of haploid species from the *E. poae* and the *E. sibirica* clades, being the latest addition (Tian et al. 2020). With the exception of the hybrid *E. disjuncta*, and the contributors of the “Lolium-associated clade” in certain hybrid species found in some *Lolium* spp., all ancestors of the described hybrid *Epichloë* spp. can be attributed to existing phylogenetic clades. For *E. disjuncta,* only one of the ancestors falls into a known clade related to endophytes of *Brachipodium* hosts (*E. typhina* or *E. sylvatica*) (Leuchtmann and Oberhofer 2013). The second ancestor was until now believed to be either extinct or not yet found.

So far, there are 20 genomes of *Epichloë* available, representing 15 species. Only the genome of *Epichloë festucae* Fl1 is assembled on a chromosome level. It consists of seven chromosomes and one mitogenome with a genome size of 35 Mb and a GC content of 43.9% (Winter et al. 2018).

There are several reports of *Epichloë* infecting grasses of the genus *Melica*. *Epichloë tembladerae* was found in *M. stuckertii* (Gentile et al. 2005), *E. melicicola* in *M. racemose* and *M. decumbens* (Moon et al. 2002) and *E. guerinii* infecting *M. transsilvanica* and *M. ciliate* (Moon et al. 2007).

*Melica uniflora*, a perennial rhizomatous cool-season grass of tribe *Meliceae* that grows in shady places in Europe, northwards to Scotland and Southwest Finland and eastwards to Moldavia (Tutin et al. 2010) was until now not reported to be a host for *Epichloë* (White and Baldwin 1992; Wilson et al. 1991).

In the “Butterberggelände” nature reserve in Bad Harzburg, Germany, several *M. uniflora* individuals showing stromata were found. The plants were sampled to isolate the fungal candidate causing the infection. Here we describe a new haploid *Epichloë* species isolated from *M. uniflora*, and report its telomere-to-telomere de novo genome assembly.

## MATERIALS AND METHODS

### Biological materials/fungal isolation

Two *M. uniflora* individuals bearing stromata were collected in June 2020 in the “Butterberggelände” nature reserve (NSG BR 004) 51° 53′ 13.5″ N, 10° 34′ 36.9″ E. This is a melic grass / beech forest characterized by stony limestone weathered soils, which are well supplied with nutrients. One plant individual was sampled with attached roots and brought to the greenhouse, while the other only consisted of aerial tissue. The grasses were identified based on the flowering tillers and identity was confirmed using DNA-based identification techniques (see below).

Endophytes were isolated from *M. uniflora* pseudostems according to “Basic Protocol 4”, described by Florea et al. (2015). Another isolation was made directly from the stroma of the greenhouse specimen by surface swab, followed by a series of subculturing steps.

### Morphological examination

Fungal structures were examined and photographed using a Zeiss Axioskop 2 plus compound microscope and an Olympus SZX 12 stereo microscope equipped with a Jenoptik ProgRes^®^ digital camera. Images were recorded using CapturePro 2.8 software (Jenoptic, Jena, Germany). Growing mycelia mounted in water, as well as slide cultures (Gams 1998) were used to illustrate fungal structures in different developmental stages. Nomarski Differential Interference Contrast (DIC) optics were used for observation and measurements. All measurements were obtaind from cultures growing on potato dextrose agar (PDA, Merck) and are given as × 1– × 2 (× 3 ± SD), with × 1 = minimum value observed, × 2 = maximum value observed, × 3 = average, and standard deviation (SD), followed by the number of measurements (n). Color changes of fungal structures formed in culture were checked using 3% potassium hydroxide (KOH) watery solution. Color codes used in the description were determined according to https://www.ral-farben.de/en/all-ral-colours.

### Growth rate studies

Growth rates were determined at various temperatures from 5 to 35 °C at 5 °C intervals in the dark. Agar disks of 4 mm diam, excised from the margin of a young PDA culture were placed onto four replicate plates of PDA, cornmeal agar (CMA, Fluka), and yeast malt agar (YM: 3 g yeast extract, 3 g malt extract, 5 g peptone, 10 g glucose, 20 g agar, 1L deionized water). The colony diameter was measured weekly for a 4 wk period.

### Confocal laser-scanning microscopy

Stromata and substromata samples were cut in approx. 0.1 cm cross sections by hand with a scalpel blade. Leaf sheath and blade samples were cut in 2 cm long subsamples. All samples were stained with Wheat Germ Agglutinin, Alexa Fluor™ 488 Conjugate (ThermoFisher Scientific, MA, USA) and Aniline Blue diammonium salt (C_37_H_32_N_5_O_9_S_3_) and eventually with propidium iodide (both: Sigma-Aldrich Chemie, Traufkirchen, Germany) as described by Becker et al. (2018). Samples were transferred to microscopic slides and embedded in staining solution for 10 min before microscopic examination. Confocal laser scanning microscopy (CLSM) was done using a Leica TCS SP8 as described in Becker et al. (2018).

### Molecular studies

DNA extraction from single host plants and seeds was performed using DNeasy^®^ Plant Mini Kit (QIAGEN, Germany) following the standard procedure according to the manufacturer. DNA extraction from individuals used for the distribution analysis was performed using the 96 well plate nexttec™ 1-Step Plant DNA Extraction kit (nexttec™ Biotechnologie, Germany) following the standard procedure provided by the manufacturer.

Genomic DNA from fungal isolates was extracted by transferring roughly 1 cm^2^ of fresh mycelium grown on PDA to lysis buffer (150 mM EDTA, 50 mM Tris–HCl, 1% sodium lauroyl sarcosine). After incubation at 70 °C for 30 min, DNA was isolated from the aqueous phase by sequential precipitations with 5 M potassium acetate and isopropanol followed by a washing step with 70% ethanol. Final DNA was resuspended in 50 μl of PCR-grade water.

High molecular weight (HMW) genomic DNA (gDNA) for genome sequencing was extracted using the modified protocol of Mayjonade et al. (2016). 2 × 15 mg of freeze-dried mycelium (grown as liquid culture in PD broth) were transferred to one 1.6 ml bead-tube filled with 140 mg ceramic beads (Ø1.4–1.6 mm) and 4 ceramic beads (Ø 2.6–2.8 mm) each. Tubes were cooled down in liquid nitrogen before and between 2 bead-beating steps for 15 s in BeadRupter (Biolabproducts, Germany) at speed 4.00. After addition of 600 µl of lysis-buffer (preheated to 65 °C) including RNase A, samples were incubated at 65 °C for 5 min until all mycelium was dissolved. Afterwards, samples were incubated at 50 °C for 30 min and mixed by inverting (20 times) every 10 min. After addition of 200 µl 5 M potassium acetate, samples were placed on ice to cool down and centrifuged at 5,000 × g for 10 min at 4 °C. Supernatant was transferred to a fresh tube and 1 vol. of binding buffer and 1:18 (v:v) of Serapure beads solution were added followed by incubation at room temperature for 10 min in a rotary shaker. Tubes were placed on a magnetic rack for 1 h, supernatant was removed and beads were washed 2 times with 1 ml of wash solution. DNA was eluted by adding 100 µl of preheated (50 °C) elution buffer. DNA was cleaned using 1 vol. of bead-solution according to the protocol by Schalamun et al. (2019). DNA concentration was measured using Qubit and integrity of the DNA was checked on a 0.5% Agarose gel.

Translation elongation factor 1-α (*tefA*), β-tubulin (*tubB*), calmodium M (*calM*) and internal transcribed spacers including 5.8S rDNA (ITS) loci were amplified using the primer pairs tef1-exon1d-1 and tef1-exon6u-1 (Craven et al. 2001), T1.1 and T1.2 (Young et al. 2005), cal-exon1d and cal-exon7u (Mc Cargo et al. 2014) and ITS5 and ITS 4 (White et al. 1990), respectively. For determination of the alkaloid profile and mating type a multiplex PCR was conducted as described by Charlton et al. (2014). PCR reactions for *tefA*, *tubB, calM,* ITS and multiplex PCR were performed in a total volume of 30 µl 1 × Green GoTaq™ Reaction Buffer, containing 3 ng DNA, 1.25 U GoTaq™ DNA Polymerase (Promega, Germany), 0.2 mM of each dNTP (Biozym Scientific, Germany), and 1 µM target-specific primers. The following thermal cycling parameters were used for both single target (*tefA* and *tubB*) and multiplex PCR: 94 °C for 1 min, then 40 cycles of 94 °C for 15 s, 56 °C for 30 s, and 72 °C for 1 min followed by 72 °C for 10 min. Cycling parameter for *calM* were as followed: 94 °C for 2 min, then 40 cycles of 94 °C for 45 s, 50 °C for 45 s, and 72 °C for 1 min followed by 72 °C for 10 min.

For confirmation of identity of the host species, PCR reactions were performed to amplify ribulose-bisphosphate carboxylase (*rbcL*) in a total volume of 30 µl 1 × Green GoTaq™ Reaction Buffer, containing 2 ng DNA, 1 U GoTaq™ DNA Polymerase (Promega), 0.3 mM of each dNTP (Biozym Scientific, Germany), and 0.5 µM each of rbcL_1_for and rbcL_1388_rev primers (Christin et al. 2008) using the following cycling parameters: 94 °C for 3 min, then 40 cycles of 94 °C for 1 min, 48 °C for 30 s, and 72 °C for 2 min followed by 72 °C for 7 min.

PCR products of *tefA* were cloned using pGEM^®^-T Easy Vector System I (Promega). For fungal isolates studied here (DSM111774, DSM111775 and DSM112488), a minimum of eight colonies per isolate containing the *tefA* sequences were sequenced with primers SP6 and T7. PCR products of ITS, *tubB, calM* and *rbcL* were sequenced using the same primers used for the PCR. Sequences obtained were assembled using CLC Main Workbench v20.0.4 (QIAGEN Aarhus A/S). Sequences obtained were deposited in GenBank under accession numbers MZ147888, MZ147889, MZ147863, MZ198217-MZ198219, MZ224334-MZ224338, MZ438661-MZ438663.

### Molecular phylogenetic analyses

In order to create the dataset for a multigene phylogeny a search was conducted using the NCBI Nucleotide database. The search resulted in 800 and 1065 sequences of tefA and *tubB*, respectively. Sequences were extracted from the XML-files, combined in two separated FASTA-files for each gene. An additional file was created containing species name, accession number, gene, strain, isolate, and sequence definition. This file was used to find matching pairs regarding species and strain/isolate. Information on allele was added manually based on the information given in the sequence definition. A total of 438 sequences could be identified showing matching pairs of *tefA* and *tubB*. Both previously created datasets were reduced to those sequences. A subset was created containing one available individual of each species, subspecies and variation based on sequence length and quality. Details of the sequences used for the alignment can be found in the table of taxa (Table 1).

**Table 1:** Table of taxa. Sequences in the *tefA* and *tubB* alignments used for the multigene phylogeny

Species	Isolate number	Allele	Host	Loc	Genbank accession No		References
					*tubB*	*tefA*	
*Claviceps purpurea*	20.1		*Secale cereale*		KP689578	HQ026480	–
*Epichloë amarillans*	E4668		*Agrostis hyemalis*	N.A	KF042042	KP689563	Schardl et al. (2013)
*E. aotearoae*	e899		*Echinopogon ovatus*	NZ	KF042049	KP689565	Schardl et al. (2013)
*E. australiensis*	AL1759f	allele 1	*Dichelachne micrantha*	NZ	MN150703	MN150705	Leuchtmann et al. (2019)
*E. australiensis*	AL1759p	allele 2	*Dichelachne micrantha*	NZ	MN150704	MN150706	Leuchtmann et al. (2019)
*E. baconii*	9707		*Agrostis tenuis*	CH	KF811579	KF811547	Ekanayake et al. (2013)
*E. brachyelytri*	E4804		*Brachyelytrum erectum*	US	KF042060	KP689564	Schardl et al. (2013)
*E. bromicola*	AL0426 2 E7561		*Thinopyrum intermedium*		KP689571	KP689559	–
*E. cabralii*	BlaTG 2	allele 1	*Bromus laevipes*		JX679191	JX679184	–
*E. cabralii*	BlaTG 2	allele 2	*Bromus laevipes*		JX679192	JX679185	–
*E. canadensis*	CWR 34	allele 1	*Elymus canadensis*	MX	KF719190	KF719188	Charlton et al. (2012)
*E. canadensis*	CWR 34	allele 2	*Elymus canadensis*	MX	KF719191	KF719189	Charlton et al. (2012)
*E. chisosa*	134	allele 1	*Stipa eminens*	US	AF457471	AF457509	Moon et al. (2004)
*E. chisosa*	134	allele 2	*Stipa eminens*	US	AF457470	AF457508	Moon et al. (2004)
*E. chisosa*	134	allele 3	*Stipa eminens*	US	AF457472	AF457510	Moon et al. (2004)
*E. coenophiala*	Greek type 1	allele 1	*Lolium arundinaceum*	GR	JX028244	JX028257	Takach et al. (2012)
*E. coenophiala*	Greek type 1	allele 2	*Lolium arundinaceum*	GR	JX028245	JX028258	Takach et al. (2012)
*E. coenophiala*	Greek type 1	allele 3	*Lolium arundinaceum*	GR	JX028246	JX028259	Takach et al. (2012)
*E. coenophiala*	e19	allele 1	*Lolium arundinaceum*		KP689577	KP689554	–
*E. coenophiala*	e19	allele 2	*Lolium arundinaceum*		KP689576	KP689566	–
*E. coenophiala*	e19	allele 3	*Lolium arundinaceum*		KP689575	KP689556	–
*E. danica*	D2 5	allele 1	*Hordelymus europaeus*	DK	JF718475	JF718528	Oberhofer and Leuchtmann (2012)
*E. danica*	D2 5	allele 2	*Hordelymus europaeus*	DK	JF718476	JF718529	Oberhofer and Leuchtmann (2012)
*E. disjuncta*	A1 1	allele 1	*Hordelymus europaeus*	IT	JF718437	JF718490	Oberhofer and Leuchtmann (2012)
*E. disjuncta*	A1 1	allele 2	*Hordelymus europaeus*	IT	JF718438	JF718491	Oberhofer and Leuchtmann (2012)
*E. disjuncta*	A4 5	allele 1	*Hordelymus europaeus*	IT	JF718440	JF718493	Oberhofer and Leuchtmann (2012)
*E. disjuncta*	A4 5	allele 2	*Hordelymus europaeus*	IT	JF718441	JF718494	Oberhofer and Leuchtmann (2012)
*E. disjuncta*	C5a 1	allele 1	*Hordelymus europaeus*	DE	JF718469	JF718522	Oberhofer and Leuchtmann (2012)
*E. disjuncta*	C5a 1	allele 2	*Hordelymus europaeus*	DE	JF718470	JF718523	Oberhofer and Leuchtmann (2012)
*E. elymi*	ATCC 201553		*Elymus virginicus*		AF062428	AF457498	–
*E. festucae*	Fl1 E894		*Festuca trachyphylla*	NZ	KF042045	KP689555	Schardl et al. (2013)
*E. festucae* var*. lolii*	15335		*Lolium perenne*	IT	KP834584	KP834548	Hettiarachchige et al. (2015)
*E. gansuensis*	E7080		*Achnatherum inebrians*	CN	KF042053	KP689495	Schardl et al. (2013)
*E. glyceriae*	E277		*Glyceria striata*	CA	KF042046	KP689560	Schardl et al. (2013)
*E. guerinii*	CBS 113029	allele 1	*Melica ciliata*	FR	EF422748	-	Moon et al. (2007)
*E. guerinii*	CBS 113029	allele 2	*Melica ciliata*	FR	EF422749	-	Moon et al. (2007)
*E. hordelymi*	A51 5	allele 1	*Hordelymus europaeus*	IT	JF718442	JF718495	Oberhofer and Leuchtmann (2012)
*E. hordelymi*	A51 5	allele 2	*Hordelymus europaeus*	IT	JF718443	JF718496	Oberhofer and Leuchtmann (2012)
*E. inebrians*	e7478		*Achnatherum inebrians*		KP689490	KP689493	–
*E. melicicola*	822	allele 1	*Melica racemosa*	ZA	AF323383	AF323404	Moon et al. (2002)
*E. melicicola*	822	allele 2	*Melica racemosa*	ZA	AF323383	-	Moon et al. (2002)
*E. mollis*	AL9924		*Holcus mollis*		KF042061	KP689567	Schardl et al. (2013)
*E. novae-zelandiae*	AL0725 2	allele 1	*Poa matthewsii*	NZ	MN013155	MN013158	Leuchtmann et al. (2019)
*E. novae-zelandiae*	AL0725 2	allele 2	*Poa matthewsii*	NZ	MN013154	MN013157	Leuchtmann et al. (2019)
*E. novae-zelandiae*	AL0725 2	allele 3	*Poa matthewsii*	NZ	MN013153	MN013156	Leuchtmann et al. (2019)
*E. schardlii*	PA 10 10	allele 1	*Poa alsodes*	US	MF156224	KT749529	Shymanovich et al. (2017)
*E. schardlii*	PA 10 10	allele 2	*Poa alsodes*	US	MF156223	KT749530	Shymanovich et al. (2017)
*E. scottii*	DSM112488		*Melica uniflora*	DE	MZ198217	MZ224336	*
*E. scottii*	DSM111774		*Melica uniflora*	DE	MZ198218	MZ224334	*
*E. scottii*	DSM111775		*Melica uniflora*	DE	MZ198219	MZ224335	*
*E. sibirica*	MTI I03		*Achnatherum sibiricum*	CN	FJ769412	FJ769418	Zhang et al. (2009)
*E. siegelii*	ATCC 74483 type e915	allele 1	*Festuca pratensis*	DE	AF308139	AF308132	Craven et al. (2001)
*E. siegelii*	ATCC 74483 type e915	allele 2	*Festuca pratensis*	DE	AF308138	AF308133	Craven et al. (2001)
*E. sinensis*	57A	allele 1	*Festuca sinensis*	CN	KX685661	KX685663	Tian et al. (2020)
*E. sinensis*	57A	allele 2	*Festuca sinensis*	CN	KX685660	KX685662	Tian et al. (2020)
*E. stromatolonga*	Chsa102		*Calamagrostis epigeios*	CN	KC463811	KC463806	–
*E. sylvatica*	GR 10156 E7368		*Brachypodium sylvaticum*		KP689573	KP689552	-
*E. sylvatica* subsp*. pollinensis*	A2 6		*Hordelymus europaeus*	IT	JF718439	JF718492	Oberhofer and Leuchtmann (2012)
*E. tembladerae*	ni 269648	allele 1	*Hordeum comosum*		KX154249	KX173839	–
*E. tembladerae*	ni 269648	allele 2	*Hordeum comosum*		KX154248	KX173838	–
*E. typhina*	9340		*Poa pratenis*	CH	KF811577	KF811545	Ekanayake et al. (2013)
*E. typhina*	E8		*Lolium perenne*	US	MF928013	MF928030	Campbell et al. (2017)
*E. typhina* subsp*. poae*	BlaTG 1		*Bromus laevipes*		JX679195	JX679188	–
*E. typhina* var*. aonikenkana*	2642		*Bromus setifolius*	AR	KF534048	KF534085	Mc Cargo et al. (2014)
*E. uncinata*	E81		*Festuca pratensis*		KF811583	KF811551	Ekanayake et al. (2013)

Locality (Loc.) is provided as ISO 3166-1 alpha-2 code. N.A is used for North America. ‘–’ in the Reference (Ref.) column indicates unpublished sequences, * marks sequences obtained during this study

Additionally, an exclusively *tubB* sequences dataset (*tubB*-only) was prepared based on the alignment published by Leuchtmann et al. (2014). The dataset was expanded by the *tubB* sequences generated here*,* both alleles from *E. alsodes* strain NY 12–14 (Shymanovich et al. 2017), *E. hybrida* strain NEA11 (Campbell et al. 2017), *E. schardlii* var. *pennsylvanica* PA 10 (Shymanovich et al. 2017), three alleles of *E. novae-zelandiae* strain AL0725 (Leuchtmann et al. 2019), and sequences of both alleles of *E. sinensis* 57A (Tian et al. 2020). DNA sequences were aligned using the online version of MAFFT v7 (Katoh et al. 2019; Kuraku et al. 2013) adopting the iterative refinement methods L-INS-i for both *tubB* and *tefA* genome regions used for the combined dataset, and FFT-NS-i for the adapted *tubB-*only dataset. Alignments were visually examined and their starts and ends were manually trimmed using AliView v. 1.26 (Larsson 2014). Phylogenetic analyses were applied using Bayesian Inference (BI), maximum-likelihood (ML) and neighbor-joining (NJ) for all datasets. The best-fit model of DNA substitution was estimated using MrModeltest v2.2 (Nylander 2004) under hierarchical likelihood ratio test (hLRT) and the Akaike Information Criterion (AIC). The general time reversible model with gamma distributed substitution rates and invariate sites (GTR + I + G) and the symmetrical model with gamma distributed substitution rates and invariate sites (SYM + I + G) were selected as the best fitting model for the combined aligned dataset, under hLRT and AIC, respectively. Substitution models for the *tubB*-only dataset selected using MrModeltest were including K80 + G (hLRT) and K80 + I + G (AIC).

For the multigene phylogeny, Bayesian analysis was performed using Metropolis Coupled Monte Carlo Markov chains (MCMCMC) based on both best fitting models settings in MrBayes v3.2 (Ronquist and Huelsenbeck 2003). The process was run for 2,000,000 generations and trees were sampled every 500 generations. A 50% majority rule consensus tree was computed only from trees of the plateau, and if, additionally, the split frequencies were below 0.01. A total of 1701 trees representing the “burn-in phase” were discarded and the remaining trees were used to infer posterior probabilities (PP) for the nodes of the majority rule consensus tree. ML and NJ analyses were conducted as previously described (Ashrafi et al. 2017). For the *tubB*-only dataset, Bayesian analysis was executed through Markov Chain Monte Carlo (MCMC) sampling as described above. The number of generations was set at 3,000,000. A total of 2401 trees were discarded as burn-in and the remaining 3600 trees were used to calculate PP of the majority rule consensus tree. The phylograms were visualized using FigTree v1.4.3 (http://tree.bio.ed.ac.uk/software/figtree). The alignments and respective phylogenetic trees were uploaded in TreeBASE under the submission number: ID 28694 (http://purl.org/phylo/treebase/phylows/study/TB2:S28694).

A third phylogenetic analysis was performed based on Benchmarking Universal Single-Copy Orthologs (BUSCO). The dataset for this analysis comprised the genomes of all haploid species in the genus *Epichloë* that are available in the NCBI database, namely *Epichloë amarillans* E57 (NCBI accession number GCA_000223075.2), *E. aotearoae* E899 (GCA_000729855.1), *E. baconii* E1031 (GCA_000729845.1), *E. brachyelytri* E4804 (GCA_000222915.1), *E. bromicola* AL0434 (GCA_001008065.1), *E. elymi* E757 (GCA_002591845.1), *E. festucae* Fl1 (GCA_003814445.1), *E. gansuensis* E7080 (GCA_000222895.2), *E. glyceriae* E277 (GCA_000225285.2), *E. inebrians* E818 (GCA_000309355.1), *E. mollis* (E3601 (GCA_000729825.1), *E. sylvatica* E7368 (GCA_001008265.1) and *E. typhina* E8 (GCA_000308955.1), as well as the genome of the new species described here (isolate DSM112488) and two outgroup species, *Claviceps paspali* C7990 (GCA_000223175.2) and *C. purpurea* C20.1 (GCA_000347355.1). BUSCO v5.2.2 (Manni et al. 2021) was used to identify conserved gene orthologs independently in each species using the closest BUSCO reference database, hypocreales_odb10. Single copy ortholog protein sequences were passed to OrthoFinder v2.5.4 (Emms and Kelly 2019), which generated a maximum likelihood species phylogeny using RAxML-NG v1.0.3 (Kozlov et al. 2019) based on 2,828 orthologs with < 5% missingness across species. Branch support values were calculated using the Shimodaira-Hasegawa algorithm in FastTree v2.1.10 (Price et al. 2010) with 1000 × resampling.

### Genome sequencing and assembly

The genome was sequenced using the previously isolated HMW gDNA following the Oxford Nanopore Technologies (ONT) Genomic DNA by Ligation protocol for the SQK-LSK110 Ligation Sequencing Kit (Version: GDE_9108_v110_revE_10Nov2020). Sequencing run was performed on a MinION 1B in a R10.3 Flow Cell for 72 h. Basecalling was performed on a NVIDIA Jetson AGX Xavier developer kit using ONT Guppy v5.0.11 and the r9.4.1 HAC model. Reads were corrected, trimmed and assembled using Canu (Koren et al. 2017) (snapshot v2.2-development + 149 changes (r10258)). Genome size was set to 38 Mb and the correctedErrorRate parameter was set to 0.039 for the assembly step.

Polishing of the genome was performed by mapping high-coverage Nanopore long read data to the assembled genome using minimap2 v2.22 (Li 2018) and Pilon v1.24 (Walker et al. 2014). The genome was submitted to GenBank under the accession numbers CP083245-CP083252 and the corresponding BioProject PRJNA756890 and BioSample SAMN20929797 numbers.

Repeat elements were identified with RepeatModeler v2.0.2a and RepeatMasker v4.1.2.p1, and their density across the genome was calculated with the ‘coverage’ function of bedtools v2.30.0.

### Distribution analysis

**Fig. 1:**
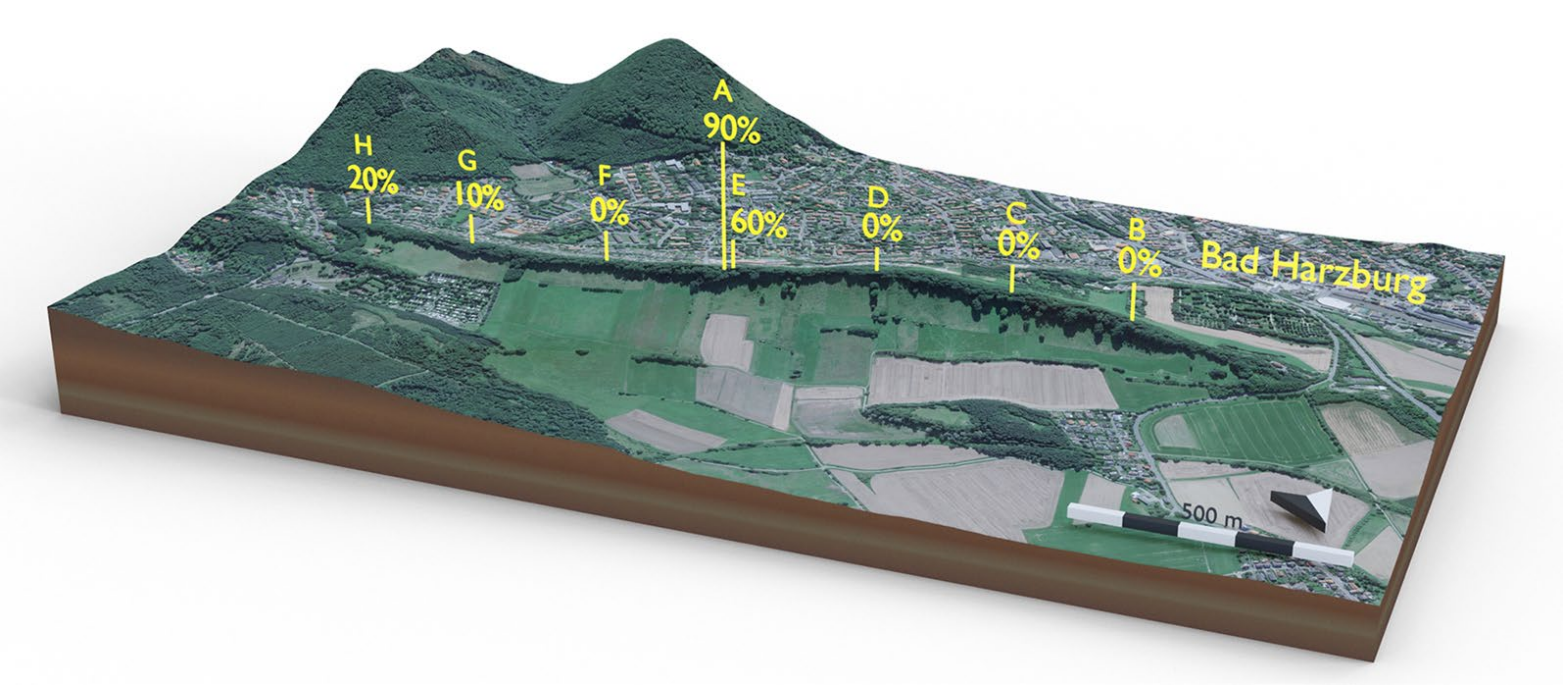
Sample sites in the nature reserve”Butterberggelände “ in Bad Harzburg, Germany. Site A had a radius of 0.5 m; sites B to H had a radius of 5 m. Stromata bearing samples were discovered at sites A and E. For distribution analysis at each site, 10 specimens with inflorescences and no visible stromata were collected. Numbers in percent indicate the infection rate at the respective site. Scale bar is 500 m. Arrow indicates north. Map was created using blender (https://www.blender.org/) and BlenderGIS PlugIn (https://github.com/domlysz/BlenderGIS). Map data ©2020 Google

To determine the distribution of the fungus studied here in the natural reserve “Butterberggelände”, *M. uniflora* grasses from seven areas evenly distributed along the reserve (sample sites B to H in Fig. 1) were collected at the end of August 2020*.* Each area had a radius of 5 m. Additionally an area with a radius of 0.5 m was sampled at the site where the initial stroma-bearing individuals had been detected (sample site A in Fig. 1). Within those areas, ten *M. uniflora* individuals with inflorescence and no visible stromata were collected and checked for the presence and mating type of *Epichloë* using *tubB* and *mtAC*/*mtBA* PCR amplification. Seeds of the samples were separated and checked for the presence of *Epichloë* using the same methodology mentioned earlier.

## Results

Two fungal strains DSM111774 and DSM111775 were isolated from surface sterilized pseudostems of the two *Melica uniflora* individuals sampled from sampling sites A and E (Fig. 1). Identity of the plant host was confirmed by *rbcL* gene sequence blast against the NCBI database.

**Fig. 2:**
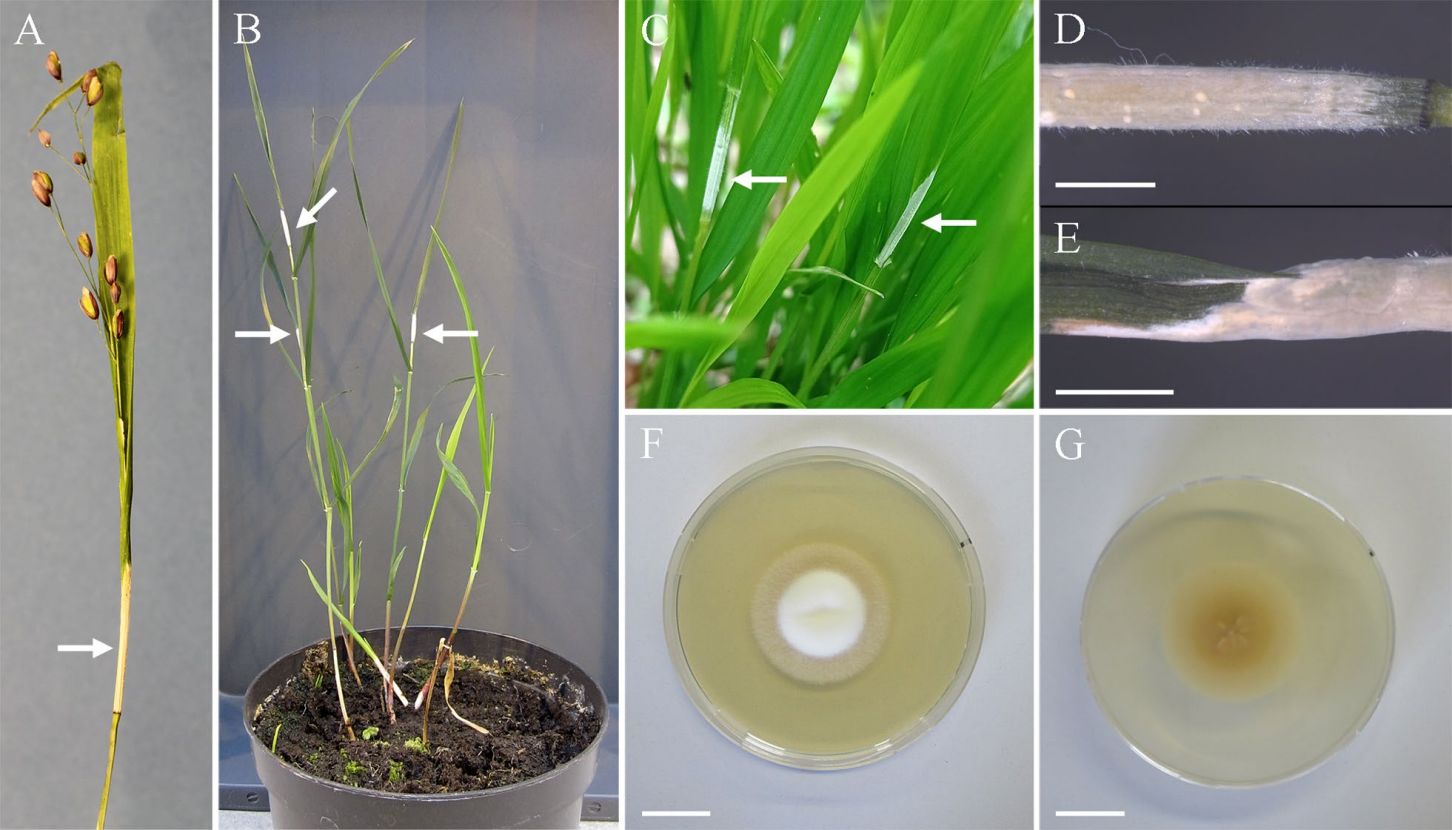
Stromata on *Melica uniflora* (indicated by arrows) and growth in culture media. **a** Partial choking on a stroma bearing specimen of *M. uniflora* collected at sample site A. **b** Daughter ramets with stromata of the specimen collected at sample site A grown in the greenhouse. **c** Stromata on *M. uniflora* at sample site E. **d**, **e** Close-up images of a stroma on a daughter ramet grown in the greenhouse. Scale bars: 3 mm. **f**, **g** Isolate growing on PDA, top view (**f**) and bottom view (**g**). Bars = 2 cm

The specimen with roots was kept in the greenhouse of the Julius Kühn Institute, Braunschweig, Germany. Aerial tissue of this individual died off, but daughter ramets emerged from the rhizome, bearing developing stromata (Fig. 2b). The fungal strain DSM112488 was isolated directly from a developing stroma.

**Fig. 3:**
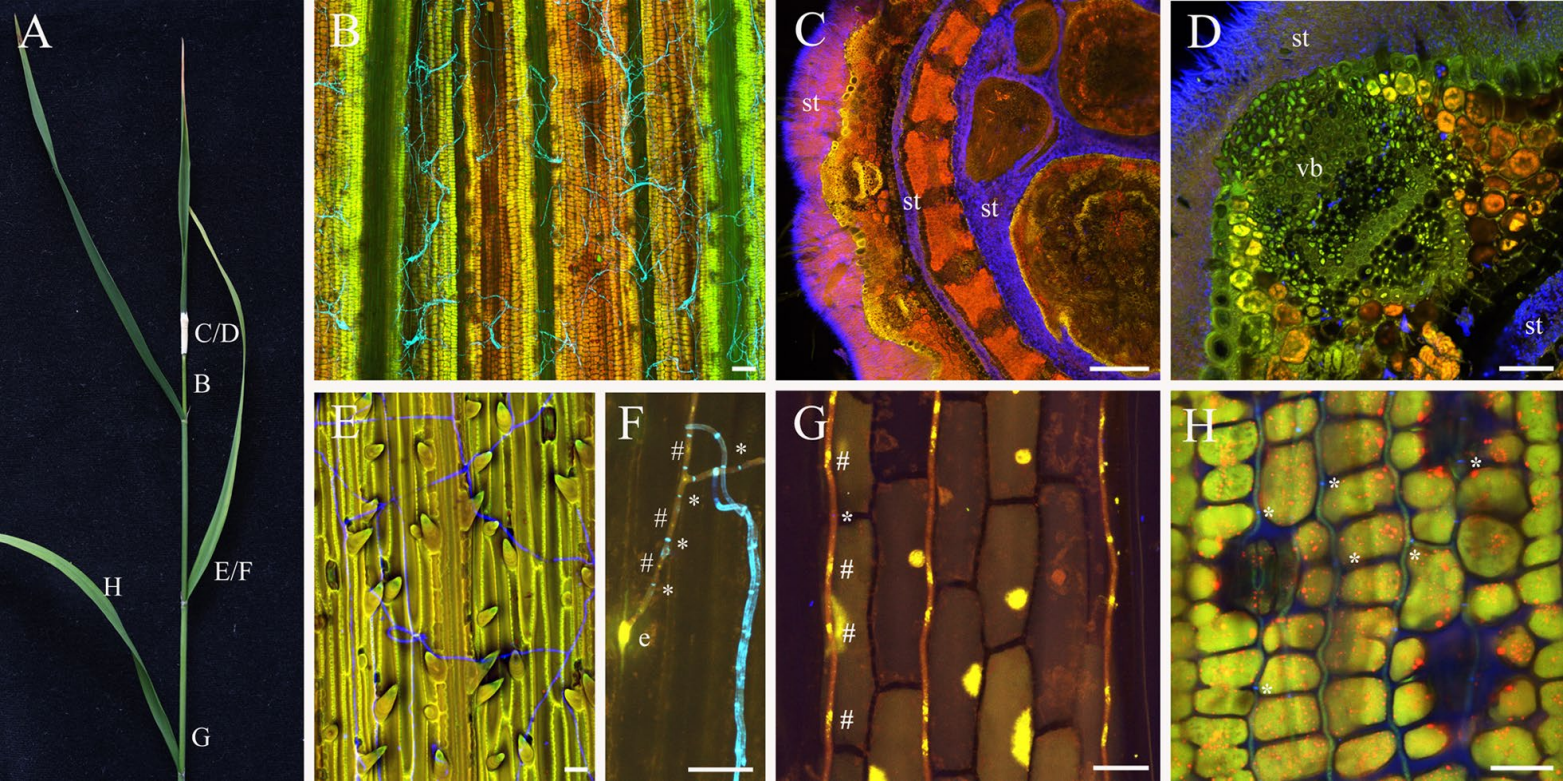
Photograph and confocal laser scanning micrographs of *Epichloë scottii* – *Melica uniflora* association. **a** Photograph of *E. scottii* stroma bearing reproductive tiller of *M. uniflora*. Letters indicate the leaf parts, where samples were taken for CLS microscopy. **b–h** confocal laser scanning micrographs. Samples were treated with aniline blue diammonium salt to stain fungal and plant cell wall β-glucans (overlay of stain and autofluorescence of cytoplasm depicted in yellow, orange pseudo colors) and wheat germ agglutinin-Alexa Fluor 488 (WGA-AF488) to stain fungal chitin (blue pseudo color) and with propidium iodide (**f**, **g**, only) to stain plant and fungal nuclei (yellow pseudo color). (**b**) epibiotic hyphae of *E. scottii* on the surface of the stem 2 cm below the base of stroma, maximum projection of 150 µm z-stack. (**c**) transverse section of unfertilized stroma tissue (st), maximum projection of 35 µm z-stack. **d** Higher magnification of (**c**) showing densely colonized vascular bundle (vb) and hyper-proliferative hyphal growth on the cuticle. **e** epibiotic hyphae of *E. scottii* on the leaf sheath. **f** epibiotic hyphae at point of exit of endobiotic hypha (e = expressorium), maximum projection of 9.4 µm z-stack. Only after several hyphal compartments are formed, chitin can be visualized by staining with WGA-AF488, depicted in blue pseudo color. **g** endobiotic hyphae of *E. scottii* in leaf sheath epidermis, maximum projection of 144 µm z-stack. **h** endobiotic hyphae of *E. scottii* in leaf blade, the channel for blue pseudocolor is overexposed to visualize chitin in septa of endobiotic hyphae, maximum projection of 12 µm z-stack. Septa in F, G, and H are marked with asterisks (*) and nuclei in (**f**) and (**g**) with hashes (#). Bars = 20 µm (**e**–**h**), 50 µm (**b**, **d**) 200 µm (**c**)

Partial as well as complete choking of the inflorescence on *Melica uniflora* was observed in the field and greenhouse (Fig. 2a–e). CLSM microscopic observations (Fig. 3) confirmed epibiotic and endobiotic growth of a fungus with similar growth phenotype as observed for other *Epichloë* species including *E. elymi* and *E. typhina* (Becker et al. 2016; Berry et al. 2021). Hyphae colonize the intercellular spaces of the plant’s aerial tissues forming a restricted hyphal net mostly parallel to the leaf’s axis, usually omitting host vascular bundle cells (Fig. 3g, h). Endobiotic hyphae formed plant exit structures, expressoria, to establish restricted epibiotic hyphal net on the leaf surface (Fig. 3b, e, f). During stroma formation of *Epichloë* spp. hyphae switch to prolific endobiotic and epibiotic growth and colonize host vascular bundles. This was also observed in the species described here (Fig. 3c, d).

### Sequence comparison and phylogenetic reconstructions

The combined dataset consisted of *tefA* (927 sites) and *tubB* (789 sites) partial sequences with a total length of 1716 base pairs (bp). The alignment comprised 65 taxa representing 33 species in the genus *Epichloë* and the species *Claviceps purpurea* as the outgroup. The DNA sequences of *tubB* and *tefA* obtained from the analyzed specimens of the *Epichloë* species studied here were identical between isolates.

**Fig. 4:**
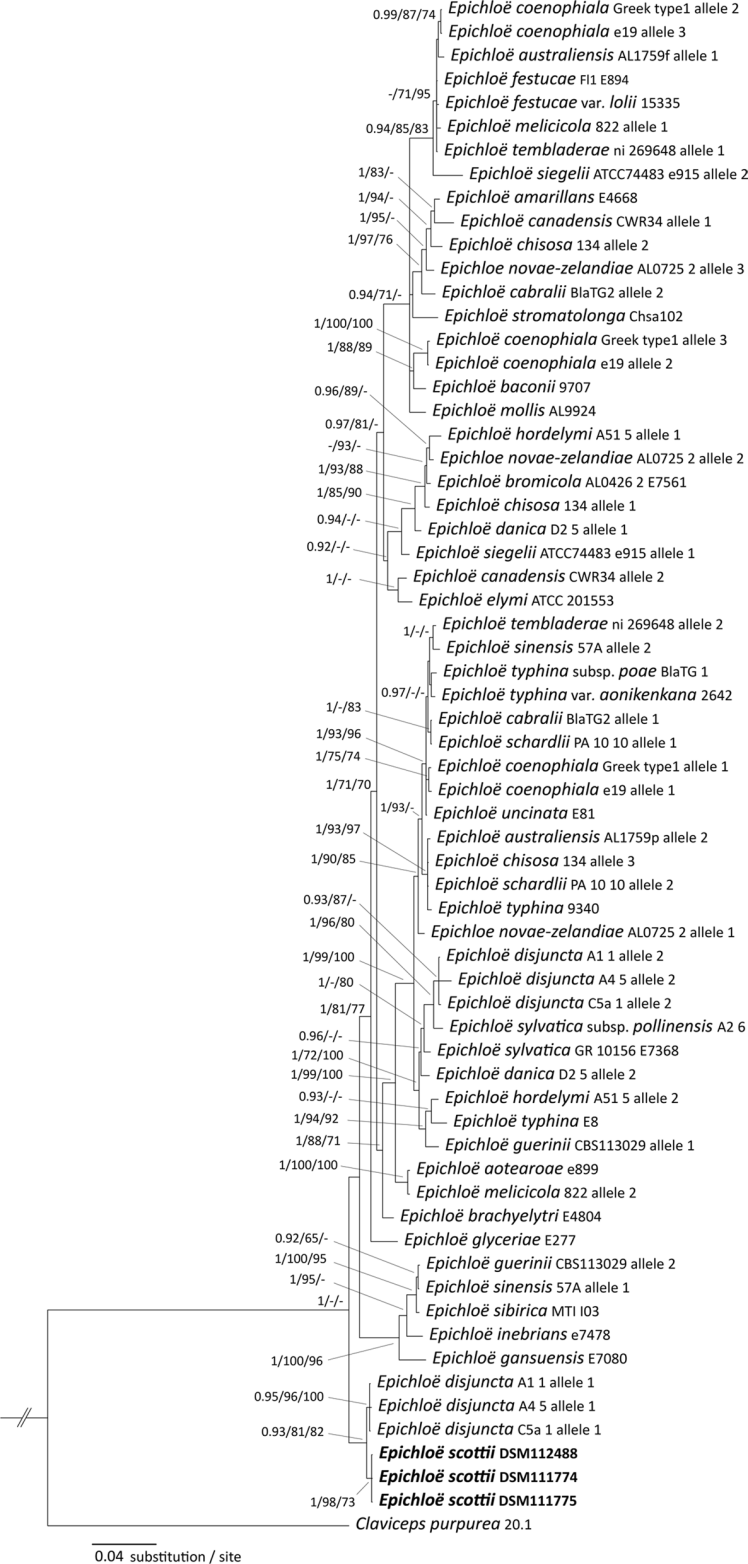
Bayesian inference of the phylogenetic relationship of the fungus described here among *Epichloë* isolates based on *tubB* and *tefA* sequences using SYM + I + G as the nucleotide substitution model. Numbers above nodes are estimates of a posteriori probability (BIpp, ≥ 0.9), and bootstrap values of maximum likelihood (ML) and neighbor-joining (NJ) (≥ 70%), respectively. The topology was rooted with the distantly related species *Calviceps purpurea*

The topologies of the phylogenetic trees were identical without any conflict in supported groupings using Bayesian inference (Fig. 4), neighbor-joining or maximum likelihood. Strains of the fungus described here were highly supported as a monophyletic species group in all analyses, and clustered with *E. disjuncta* allele 1 in a well-supported clade. Tree topologies of BI analyses conducted based on substitution models were identical. Comparison of *tubB* and *tefA* between the strains studied here and *E. disjuncta* allele 1 showed a 100% identity for *tubB* and 99.2% and 99.0% identity for *tefA* of *E. disjuncta* strains A1_1 and C5a_1 and strain A4_5, respectively.

The *tubB*-only alignment consisted of 211 sequences with 599 sites. The sequences analyzed represented 39 known *Epichloë* species, 11 specimens of *Epichloë* spp., as well the *Epichloë* species described here. This analysis also showed that the strains examined here formed a highly supported clade together with *E. disjuncta*, providing sufficient phylogenetic resolution to distinguish this group from all other *Epichloë* groupings (Fig. 8).

**Fig. 5:**
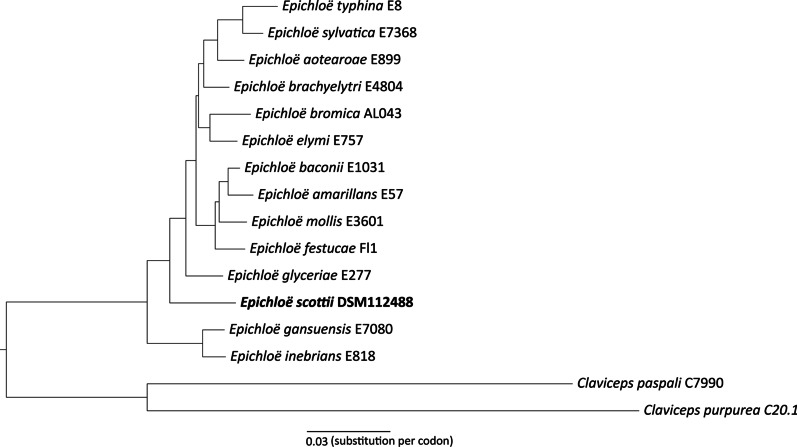
Multi-gene phylogeny of haploid species of the genus *Epichloë*. A maximum likelihood phylogeny of all haploid species in the genus *Epichloë* with an available genome reference was built using 2,828 single copy protein orthologs and rooted on two species in the outgroup genus *Claviceps*. All branches have support values of 1

The phylogentic analysis based on 2,828 BUSCO single copy protein orthologs showed an early branching of the species described here after branching of the *E. gansuensis* and *E. inebrians* clade and before *E. glyceriae* (see Fig. 5). Based on the available data *E. scottii* forms an independent lineage within the genus *Epichloë*.

## TAXONOMY

***Epichloë scottii*** T. Thünen, Y. Becker, M.P. Cox & S. Ashrafi, **sp. nov.**

MycoBank No.: MB840953.

Figure 6

*Etymology*: In honor of Barry Scott recognizing his outstanding works on the genus *Epichloë*.

*Diagnosis*: *Epichloë scottii* is characterized by small-sized conidia, short-length conidiogenous cells and moderate growth rate. The fungus develops conidiogenous cells (14.1 ± 2.8 µm) that are shorter that those of *E. disjuncta* (33.8 ± 7.3 µm). Sequence comparison of *tubB* and *tefA* between the *E. scottii* and *E. disjuncta* allele 1 showed a 100% identity for *tubB* and 99.2% and 99.0% identity for *tefA* of *E. disjuncta* strains A1_1 and C5a_1 and strain A4_5, respectively.

*Type*: **Germany**: Bad Harzburg, a dried biologically inert culture on PDA, originating from a single conidium of an immature individual stroma from “Butterberggelände” nature reserve developed on *Melica uniflora* maintained in a greenhouse, Sept. 2020, isol. *S. Ashrafi & T. Thünen*, BB005 (B 70 0,100,236 – holotype; dried plant materials bearing the immature stroma used for fungal isolation); DSM112488 – isotype culture. GenBank accession nos.: ITS: MZ147888; *tefA*: MZ224335; *tubB*: MZ198219; *calM*: MZ438661.

*Additional material examined:* **Germany**: Bad Harzburg, isolated from surface sterilized leaves of *Melica uniflora*, May 2019. isol. *T. Thünen* (DSM111774), GenBank: MZ147889 (ITS); MZ224334 (*tefA*); MZ198218 (*tubB*); MZ438662 (*calM*); *ibid*. (DSM111775) GenBank: MZ147863 (ITS); MZ224335 (*tefA*); MZ198219 (*tubB*); MZ438663 (*calM*).

**Fig. 6:**
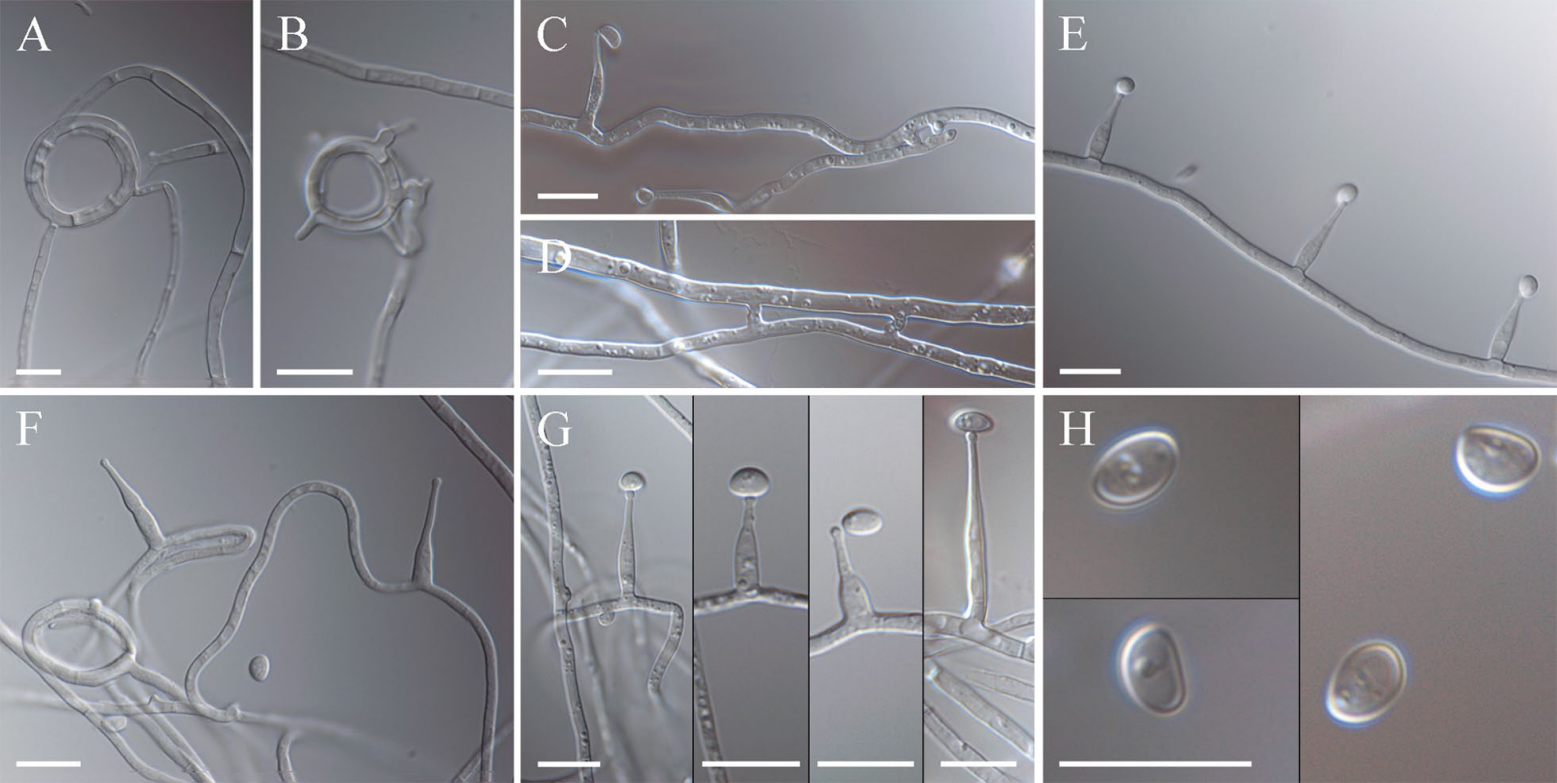
Micrographs of *Epichloë scottii* on potato dextrose agar. **a**, **b** Fungal growth and formation of coiling hyphae. **c, d** Hyphal anastomosis. **e** developing hyphae bearing conidiogenous cells and conidia. **f** Growing hyphae forming ring, conidiogenous cells arising from hyphae and conidia, **g** details of conidiogenous cells bearing conidia. **h** conidia. Bars = 10 µm

*Description*: Infested plants bearing immature stromata. *Stromata* cylindrical, variable in size, 13–29 mm long, white to lemon-yellow with age. *Colonies* moderately growing, on PDA at 20 °C reaching 7–8 mm diam (7 d), 14–16 mm diam (14 d), and 26–27 mm diam (21 d); optimum temperature for growth 20 °C; at 5 °C 1 mm (21 d), at 30 °C 3–4 mm (21 d). Optimum temperature for growth on other examined culture media at 20 °C, reaching 30–31 mm diam (CMA, after 21 d), 38–32 mm (YM, after 21 d); no growth observed at 35 °C. Colonies on PDA elevated centrally, surface smooth with dense aerial mycelium, cottony white in the central part to pale creamy at the margin, margins wide and flattened, reverse natural yellow (RAL 095 85 50) in the central part to asparagus-yellow (RAL 095 80 30) at the margin, no exudates, no medium staining (Fig. 2f, g). *Hyphae* hyaline, thin-walled, septate, forming strands or coils, occasionally anastomosed, bearing the conidiogenous cells. *Conidiogenous cells* enteroblastic, arising solitary from hyphae, hyaline, cylindrical at the base, gradually tapering towards the apex, separated by a basal septum, variable in length, 8.2–22.6 µm (14.1 ± 2.8) long and 1.5–3.5 µm (2.5 ± 0.4) wide at the base (*n* = 65). *Conidia* ellipsoid to ovoid, hyaline, smooth, aseptate, 3.5–5.6 × 2.4–3.6 µm (4.5 ± 0.4 × 3.0 ± 0.25) (*n* = 85) (Fig. 6).

*Host*: Only from *Melica uniflora.*

*Distribution:* At the end of August 2020, 80 individuals of *M. uniflora* with inflorescences but no visible stromata were collected at eight sites along a transect in the “Butterberggelände” nature reserve (Fig. 1). The survey resulted in discovery of 18 M*. uniflora* individuals infected with *Epichloë scottii*. The distribution is shown in Fig. 1 with the numbers showing the percentage infection rate observed at each sample site. Beyond the initial sample sites A and E, infected *M. uniflora* was only observed at sites G (10% infection rate) and H (20% infection rate). Because sampling was conducted quite late in the year, the majority of seeds had already fallen. Only four fully developed seeds were recovered from infected individuals. Sequencing results of the seed samples confirmed the presence of *E. scottii*.

*Discussion*: According to phylogenetic inference (Fig. 4), *E. scottii* is closely related to but distinct from *E. disjuncta*. Both taxa form moderately growing colonies on PDA. *Epichloë scottii* develops ellipsoid to ovoid and small size conidia (4.5 × 3.0 µm) whereas *E. disjuncta* forms medium-sized conidia (6.9 × 2.7 µm) that are lunate to reniform and often bear an apiculum-like bulge at the base. They also differ in the length of the conidiogenous cells. *Epichloë scottii* develops conidiogenous cells (14.1 ± 2.8 µm) that are shorter that those of *E. disjuncta* (33.8 ± 7.3 µm). Although *E. scottii* was originally isolated from *M. uniflora*, *E. disjuncta* was reported from *Hordelymus europaeus* (Leuchtmann and Oberhofer 2013). Among non-hybrid species studied by Leuchtmann and Oberhofer (2013), *E. scottii* and *E. sylvatica* subsp. *pollinensis* form conidia and conidiogenous cells that are morphometrically similar, however the difference between these species is strongly supported by sequence and genomic comparison (Figs. 4, 5).

### Genome sequencing and assembly

**Fig. 7:**
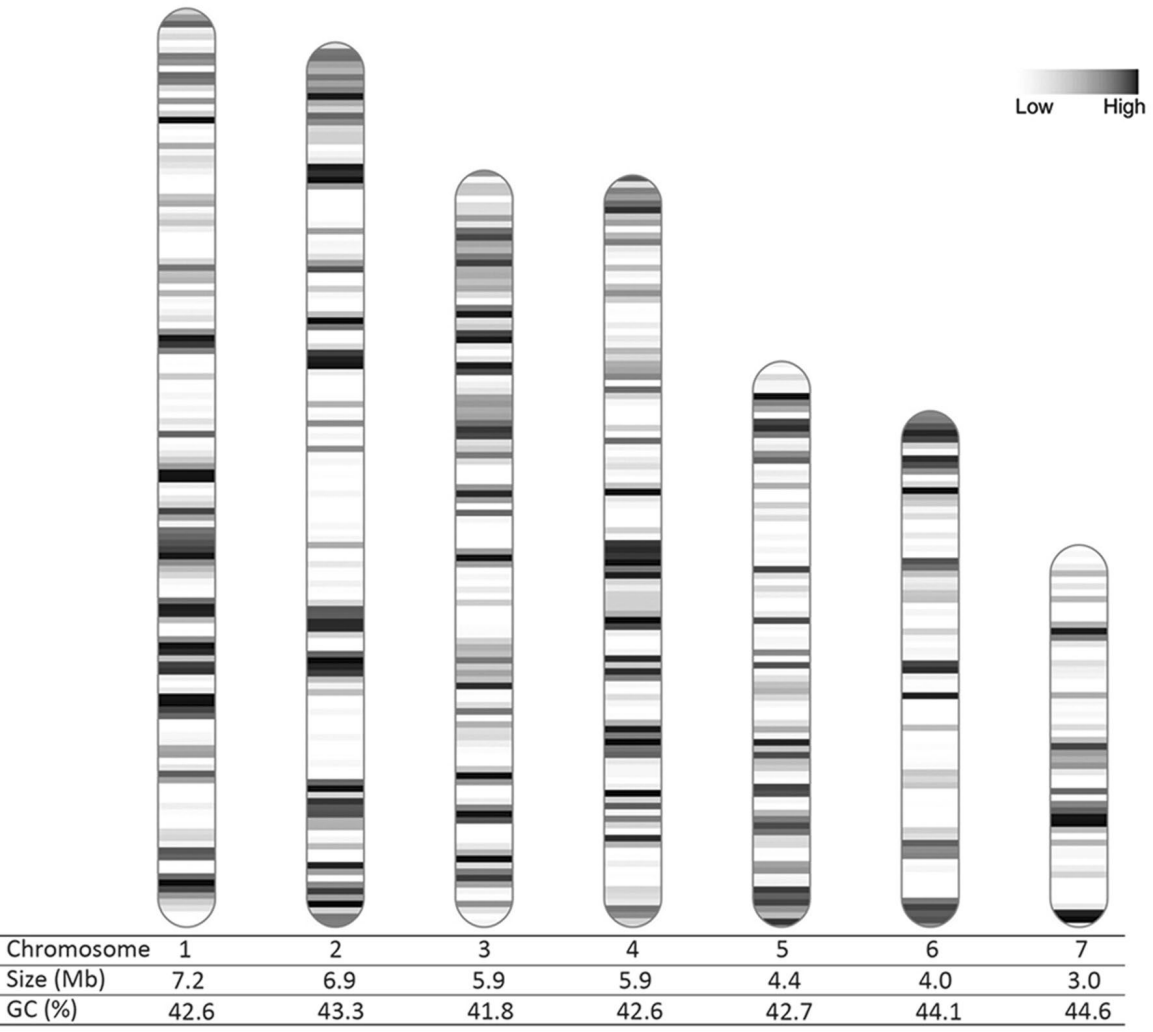
Karyogram of the seven chromosomes of *Epichloë scottii*. Individual size and GC content is given below each chromosome. Grayscale indicates the density of repeat elements from low (white) to high (back)

**Fig. 8:**
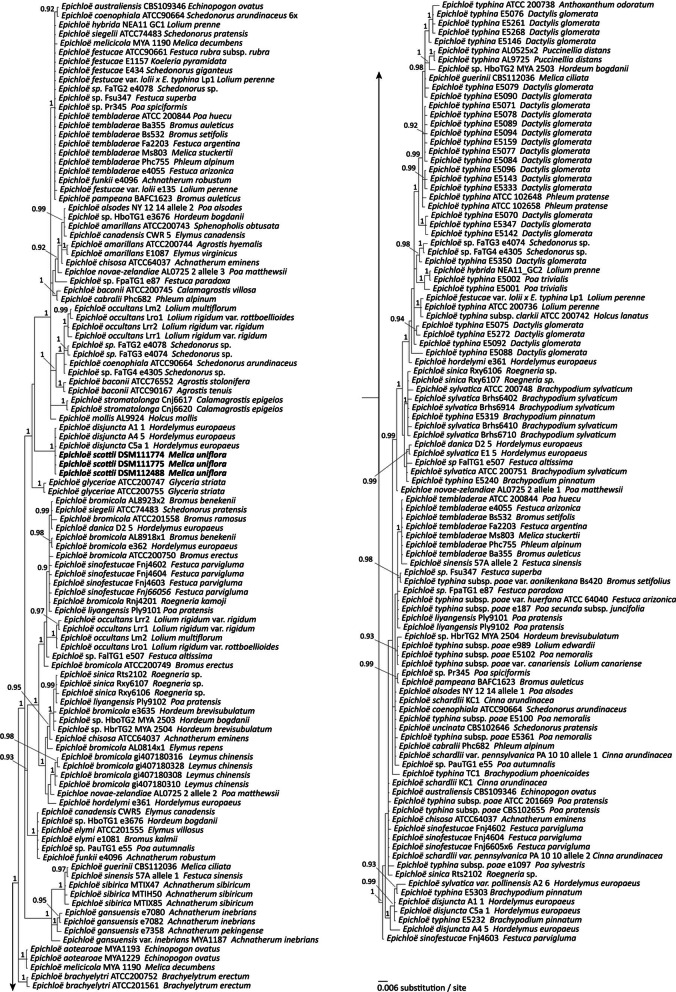
Bayesian inference of the phylogenetic relationship of *tubB* sequences of the taxa among *Epichloë* using K80 + I + G as nucleotide substitution model. Numbers above nodes are estimates of a posteriori probability (BIpp, ≥ 0.9). The strains studied here are in Bold. The tree was rooted as midpoint. Host species are provided after each strain

Initial assembly of the genome resulted in 22 contigs. Six contigs resembled complete chromosomes with telomeres on both ends. Two contigs, each with one telomere showed ribosomal DNA (rDNA) repeats at the broken ends. These two contigs therefore represent a single chromosome broken on the rDNA locus and were joined together. In order to clean up the rDNA locus two partial rDNA units at the flanks of the rDNA locus were identified, together with two complete rDNA repeats in between. One contig was identified as a broken copy of the rDNA locus and could therefore be excluded. The mitochondrial DNA (mtDNA) was present in one contig, which the assembler had identified as a circular DNA. The sequence was reordered to start at the canonical mtDNA start site. The contig contained two copies of the mtDNA genome, so the first copy was retained and the second copy deleted. The assembler identified 11 contigs as bubbles. Mapping of these contigs to the chromosomes identified high identity matches and the contigs could therefore be excluded. One small contig (3510 bp) could not be matched to the other contigs. An NCBI blast search showed that this contig is a contaminant from *Escherichia coli*, likely part of a cloning vector. This contig can therefore be safely excluded, as it is not an *Epichloë* sequence. Telomeres were trimmed to the nearest canonical repeat (TAACCC) at both ends of each chromosome. Chromosomes were sorted by length from largest to smallest. The overall length of the genome after the polishing step is 37.4 Mb distributed over seven chromosomes and one mitogenome. The overall GC content of the chromosomes is 42.9%. The length of the individual chromosomes as well as their GC content can be found in Fig. 7. The length of the mitogenome is 94,060 bp and its GC content 27.7%.

### Genetic variation

All isolates showed the same genotype regarding key genes required for alkaloid production and mating type idiomorphs (Table 2). No genes involved in the biosynthesis of ergot alkaloids and lolines could be detected by Multiplex PCR according to the method of Charlton et al. (2014). Only two indole-diterpene genes (*idtF* and *idtQ*) showed a PCR fragment of the expected size. Regarding peramine, all strains were positive for the *perA* 5′ region and the *perA* reductase domain, but the *perA* T2 domain was not detected. PCR markers for determination of the mating-type identified all strains as mating-type B. Presence and absence of genes involved in the alkaloid biosynthesis could be confirmed by crosscheck with the genome assembly of *E. scottii*.

**Table 2:** Alkaloid profile and mating type of *Epichloë scottii*

Alkaloid/mating type	Gene	*E. scottii* DSM112488	*E. scottii* DSM111774	*E. scottii* DSM111775
Ergot alkaloids	*dmaW*	−	−	−
	*easC*	−	−	−
	*easA*	−	−	−
	*cloA*	−	−	−
	*lpsB*	−	−	−
Indole-diterpenes	*idtG*	−	−	−
	*ltmJ*	−	−	−
	*idtQ*	+	+	+
	*idtF*	+	+	+
	*idtK*	−	−	−
Lolines	*lolC*	−	−	−
	*lolA*	−	−	−
	*lolO*	−	−	−
	*lolP*	−	−	−
Peramine	*perA-*5	+	+	+
	*perA-*T2	−	−	−
	*perA-*R	+	+	+
Mating type	*mtAC*	−	−	−
	*mtBA*	+	+	+

Genes for ergot alkaloid, indole-diterpene and loline biosynthesis are displayed in order of their involvement in the corresponding biosynthesis pathway. For peramine, which is encoded by a single gene, gene fragments are ordered from the 5′ to 3′ end of each gene part

## DISCUSSION

Here we report the first occurrence of an *Epichloë* endophyte in *Melica uniflora*. Comparative morphological and molecular phylogenetic evidences indicates it is a new species, given the name *Epichloë scottii* here*.* Variation in *tubB* and *tefA* sequences compared to other haploid *Epichloë* species strongly supported that *E. scottii* is a distinct and monophyletic species in the genus.

Phylogenetic analysis and sequence comparison suggests that *E. scottii* is one of the progenitor species *of E. disjuncta* (Leuchtmann and Oberhofer 2013), formerly known as *Neotyphodium* sp. HeuTG-3, and first described by Oberhofer and Leuchtmann (2012). *Epichloë disjuncta* is a hybrid *Epichloë* species isolated from the European woodland grass *Hordelymus europaeus*. *Melica uniflora* and *H. europaeus* share the same habitat. In a survey of 44 *Querco-Fageta* woods north of the Harz Mountains in Germany, *H. europaeus* could be identified in 16 areas, all of which harbor *M. uniflora* as well (Zacharias 1993). The second ancestral species of *E. disjuncta* is related to endophytes of *Brachypodium* hosts (*E. typhina* or *E. sylvatica*) (Leuchtmann and Oberhofer 2013). In the survey of Zacharias (1993), *B. sylvaticum* was identified in 43 of the 44 woods. This places both hosts of the putative ancestral species and the host of the resulting hybrid in the same habitat.

A third phylogenetic analysis based on a total of 2,828 single copy protein orthologs was conducted. Accordingly *E. scottii* was placed as an early branching species within the genus *Epichloë*. The genus of the host of *E. scottii, Melica,* is itself an early branching genus within the subfamily *Pooidae* (Saarela et al. 2018) suggesting a co-evolution of fungus and its host as described by Schardl et al. (2008). Sequencing results of PCR products showed only single alleles of the genes *tubB*, *tefA*, *calM* and the ITS region in all isolates. Therefore, *E. scottii* is considered a haploid *Epichloë* species. The BUSCO results from the genome analysis labeled almost all genes (> 99.7%) as single copy, also supporting this new species being haploid. The size of the conidia, which is comparable to other haploid *Epichloë* species (Kuldau et al. 1999) is another strong indicator for the haploid nature of *E. scottii.*

*Epichloë* species are heterothallic. For successful fertilization of the stromata they require spermatia or ascogonia from individuals of the opposite mating type, which are transmitted via vectors such as female flies of the genus *Botanophila* (Bultman and Leuchtmann 2003). No stromata on *M. uniflora* discovered during this study showed any sign of fertilization (no sign of embedded perithecia). Molecular analysis of all *E. scottii* isolates and of individuals found in the infected grasses identified them as mating type B. No individuals of mating type A have been observed in this population so far. This explains the lack of fertilized stromata.

No genes for the biosynthesis of ergot alkaloids and lolines could be detected. As the presence of these marker genes is highly correlated to alkaloid production *in planta* (Charlton et al. 2014), *E. scottii* is unlikely to produce either of these alkaloids. Only two genes involved in the biosynthesis of indole-diterpenes could be detected (*idtQ* and *idfF*): *idtG*, the first gene involved in the biosynthesis pathway (Schardl et al. 2013), is missing which makes it unlikely that *E. scottii* is able to produce any indole-diterpenes. For peramine, the marker for the T2 domain is missing. Preliminary results of the draft genome analysis showed a deletion of 2130 bp, which includes the T2 domain but also the M domain and the C-terminal subunit of the A2 domain. Due to this, neither the selection and activation of the arginine substrate, which requires the A2 C-subunit, nor the tethering or methylation of the activated arginyl residue, which requires the T2 and M domains, can be performed by the encoded protein (Tanaka et al. 2005). Based on this, the fungus described here is most likely not able to produce peramine or any other pyrrolopyrazine product.

In addition to horizontal transmission, most *Epichloë* endophytes can transmit vertically via the seeds of their hosts. DNA extraction from seeds of infected *M. uniflora* individuals, followed by PCR and sequencing showed the presence of *E. scottii*. This suggests that *E. scottii* is capable of vertical transmission. Further investigations, including microscopy of seeds of *E. scottii* infected plants is necessary to verify these findings.

The original host of *E. scottii* strain DSM111774 was brought to the greenhouse where it developed several daughter ramets, which subsequently all developed stromata. This shows that the endophyte is capable of transmission via rhizomes. For rhizomatous *Festuca ruba*, clonal growth via rhizomes can reach up to 220 m (Harberd 1961). Sample sites A and E were only 15 m apart, which makes it possible that *E. scottii* strains DSM111774 and DSM111775 originated from the same host individual.

This study showed that *E. scottii* can be transmitted vertically via clonal growth of its host and seeds. It further develops stromata in its host plants, indicating horizontal transmission of the fungus. However due to the lack of the opposite mating type development of ascospores and sexual life-cycle were not observed. Collectively, this evidence suggests *E. scottii* is a pleiotropic symbiont (Schardl et al. 1997), which can be classified as a Type II endophyte according to Clay and Schardl (2002).

## CONCLUSION

In this study we described a new, haploid and stroma forming species within the genus *Epichloë*, *Epichloë scottii* sp. nov. which was isolated from *Melica uniflora* growing in Bad Harzburg, Germany. Phylogenic analysis revealed *E. scottii* as the unknown ancestor species of the hybrid *E. disjuncta* and placed it as an early branching species within the genus *Epichloë*. We further released a telomere-to-telomere de novo assembly of all seven chromosomes and the mitogenome of *E. scottii*.

## Data Availability

All sequences generated during this study have been submitted to GenBank. Alignments and phylogenetic trees have been submitted to TreeBase and can be accessed via this link: http://purl.org/phylo/treebase/phylows/study/TB2:S28694

## Appendix A

See Fig. 8.

## Abbreviations

AIC: Akaike information criterion
BI: Bayesian inference
Bipp: Bayesian inference posteriori probability
bp: Base pairs
BUSCO: Benchmarking Universal Single-Copy Orthologs
calM: Calmodium M
CLSM: Confocal laser scanning microscope
CMA: Cornmeal agar
DIC: Differential interference contrast
dNTP: Desoxy nucleoside triphosphate
e: Expressorium
GC content: Guanine-cytosine content
gDNA: Genomic DNA
GTR + I + G: General time reversible model with gamma distributed substitution rates and invariate sites
HAC: High accuracy
hLRT: Hierarchical likelihood ratio test
HMW: High molecular weight
ITS: Internal transcribed spacer
K80 + G: Kimura 2-parameter with Gamma distributed substitution rate
K80 + I + G: Kimura 2-parameter with Gamma distributed substitution rate and invariate sites
Mb: Megabases
MCMC: Markov Chain Monte Carlo
MCMCMC: Metropolis coupled Monte Carlo Markov chains
ML: Maximum-likelihood
mtDNA: Mitochondrial DNA
NJ: Neighbor-joining
ONT: Oxford nanopore technologies
PCR: Polymerase chain reaction
PD broth: Potato dextrose broth
PDA: Potato dextrose agar
PP: Posterior probabilitiy
RAxML-NG: Randomized axelerated maximum likelihood—next generation
rbcL: Ribulose bisphosphate carboxylase large chain
rDNA: Ribosomal DNA
SD: Standard deviation
SYM + I + G: Symmetrical model with gamma distributed substitution rates and invariate sites
tefA: Translation elongation factor 1-α
tubB: β-Tubulin
vb: Vascular bundle
WGA-AF488: Wheat germ agglutinin-Alexa Fluor 488
YM: Yeast malt agar

## References

[CR1] Ashrafi S, Helaly S, Schroers H-J, Stadler M, Richert-Poeggeler KR, Dababat AA, Maier W (2017). *Ijuhya vitellina* sp. Nov., a novel source for chaetoglobosin A, is a destructive parasite of the cereal cyst nematode *Heterodera filipjevi*. PLoS ONE.

[CR2] Becker M, Becker Y, Green K, Scott B (2016). The endophytic symbiont *Epichloë festucae* establishes an epiphyllous net on the surface of *Lolium perenne* leaves by development of an expressorium, an appressorium-like leaf exit structure. New Phytol.

[CR3] Becker Y, Green K, Scott B, Becker M (2018). Artificial inoculation of *Epichloë festucae* into *Lolium perenne*, and visualisation of endophytic and epiphyllous fungal growth. Bio-Protoc.

[CR4] Berry D, Lee K, Winter D, Mace W, Becker Y, Nagabhyru P et al (2021) Cross-species transcriptomics comparing the benign asexual and antagonistic pre-sexual developmental stages of grass-endophytic *Epichloë* fungi. Under review

[CR5] Bultman TL, Leuchtmann A (2003). A test of host specialization by insect vectors as a mechanism for reproductive isolation among entomophilous fungal species. Oikos.

[CR6] Campbell MA, Tapper BA, Simpson WR, Johnson RD, Mace W, Ram A (2017). *Epichloë hybrida*, sp. Nov., an emerging model system for investigating fungal allopolyploidy. Mycologia.

[CR7] Charlton ND, Craven KD, Mittal S, Hopkins AA, Young CA (2012). *Epichloë canadensis*, a new interspecific epichloid hybrid symbiotic with Canada wildrye (*Elymus canadensis*). Mycologia.

[CR8] Charlton ND, Craven KD, Afkhami ME, Hall BA, Ghimire SR, Young CA (2014). Interspecific hybridization and bioactive alkaloid variation increases diversity in endophytic *Epichloë* species of *Bromus laevipes*. FEMS Microbiol Ecol.

[CR9] Christin P-A, Besnard G, Samaritani E, Duvall MR, Hodkinson TR, Savolainen V, Salamin N (2008). Oligocene CO_2_ decline promoted C4 photosynthesis in grasses. Curr Biol.

[CR10] Clay K, Schardl C (2002). Evolutionary origins and ecological consequences of endophyte symbiosis with grasses. Am Nat.

[CR11] Craven KD, Blankenship JD, Leuchtmann A, Hignight K, Schardl CL (2001). Hybrid fungal endophytes symbiotic with the grass *Lolium pratense*. Sydowia.

[CR12] Craven KD, Hsiau PTW, Leuchtmann A, Hollin W, Schardl CL (2001). Multigene phylogeny of *Epichloë* species, fungal symbionts of grasses. Ann Mo Bot Gard.

[CR13] Ekanayake PN, Rabinovich M, Guthridge KM, Spangenberg GC, Forster JW, Sawbridge TI (2013). Phylogenomics of fescue grass-derived fungal endophytes based on selected nuclear genes and the mitochondrial gene complement. BMC Evol Biol.

[CR14] Emms DM, Kelly S (2019). OrthoFinder: phylogenetic orthology inference for comparative genomics. Genome Biol.

[CR15] Florea S, Schardl CL, Hollin W (2015) Detection and isolation of *Epichloë* species, fungal endophytes of grasses. In: Current protocols in microbiology. Wiley. doi:10.1002/9780471729259.mc19a01s38. 10.1002/9780471729259.mc19a01s38 26237108

[CR16] Gams W (eds) (1998) CBS course of mycology. 4th ed. Centraalbureau voor Schimmelcultures, Baarn

[CR17] Gentile A, Rossi MS, Cabral D, Craven KD, Schardl CL (2005). Origin, divergence, and phylogeny of epichloë endophytes of native Argentine grasses. Mol Phylogenet Evol.

[CR18] Harberd DJ (1961). Observations on population structure and longevity of *Festuca rubra* L. New Phytol.

[CR19] Hettiarachchige IK, Ekanayake PN, Mann RC, Guthridge KM, Sawbridge TI, Spangenberg GC, Forster JW (2015). Phylogenomics of asexual *Epichloë* fungal endophytes forming associations with perennial ryegrass. BMC Evol Biol.

[CR20] Katoh K, Rozewicki J, Yamada KD (2019). MAFFT online service: multiple sequence alignment, interactive sequence choice and visualization. Brief Bioinform.

[CR21] Koren S, Walenz BP, Berlin K, Miller JR, Bergman NH, Phillippy AM (2017). Canu: scalable and accurate long-read assembly via adaptive k -mer weighting and repeat separation. Genome Res.

[CR22] Kozlov AM, Darriba D, Flouri T, Morel B, Stamatakis A (2019). RAxML-NG: a fast, scalable and user-friendly tool for maximum likelihood phylogenetic inference. Bioinformatics.

[CR23] Kuldau GA, Tsai H-F, Schardl CL (1999). Genome sizes of Epichloë species and anamorphic hybrids. Mycologia.

[CR24] Kuraku S, Zmasek CM, Nishimura O, Katoh K (2013). aLeaves facilitates on-demand exploration of metazoan gene family trees on MAFFT sequence alignment server with enhanced interactivity. Nucleic Acids Res.

[CR25] Larsson A (2014). AliView: a fast and lightweight alignment viewer and editor for large datasets. Bioinformatics.

[CR26] Leuchtmann A, Oberhofer M (2013). The *Epichloë* endophytes associated with the woodland grass *Hordelymus europaeus* including four new taxa. Mycologia.

[CR27] Leuchtmann A, Bacon CW, Schardl CL, White JF, Tadych M (2014). Nomenclatural realignment of *Neotyphodium* species with genus *Epichloë*. Mycologia.

[CR28] Leuchtmann A, Young CA, Stewart AV, Simpson WR, Hume DE, Scott B (2019). *Epichloe novae-zelandiae*, a new endophyte from the endemic New Zealand grass *Poa matthewsii*. N Z J Bot.

[CR29] Li H (2018). Minimap2: pairwise alignment for nucleotide sequences. Bioinformatics.

[CR30] Manni M, Berkeley MR, Seppey M, Simão FA, Zdobnov EM (2021). BUSCO update: novel and streamlined workflows along with broader and deeper phylogenetic coverage for scoring of eukaryotic, prokaryotic, and viral genomes. Mol Biol Evol.

[CR31] Mayjonade B, Gouzy J, Donnadieu C, Pouilly N, Marande W, Callot C (2016). Extraction of high-molecular-weight genomic DNA for long-read sequencing of single molecules. Biotechniques.

[CR32] Mc Cargo PD, Iannone LJ, Vignale MV, Schardl CL, Rossi MS (2014). Species diversity of *Epichloë* symbiotic with two grasses from southern Argentinean Patagonia. Mycologia.

[CR33] Moon CD, Miles CO, Jarlfors U, Schardl CL (2002). The evolutionary origins of three new *Neotyphodium* endophyte species from grasses indigenous to the southern hemisphere. Mycologia.

[CR34] Moon CD, Craven KD, Leuchtmann A, Clement SL, Schardl CL (2004). Prevalence of interspecific hybrids amongst asexual fungal endophytes of grasses. Mol Ecol.

[CR35] Moon CD, Guillaumin J-J, Ravel C, Li C, Craven KD, Schardl CL (2007). New *Neotyphodium* endophyte species from the grass tribes Stipeae and Meliceae. Mycologia.

[CR36] Nylander JA (2004) MrModeltest v2. Program distributed by the author

[CR37] Oberhofer M, Leuchtmann A (2012). Genetic diversity in epichloid endophytes of *Hordelymus europaeus* suggests repeated host jumps and interspecific hybridizations. Mol Ecol.

[CR38] Price MN, Dehal PS, Arkin AP (2010). FastTree 2—approximately maximum-likelihood trees for large alignments. PLoS ONE.

[CR39] Ronquist F, Huelsenbeck JP (2003). MrBayes 3: Bayesian phylogenetic inference under mixed models. Bioinformatics.

[CR40] Saarela JM, Burke SV, Wysocki WP, Barrett MD, Clark LG, Craine JM (2018). A 250 plastome phylogeny of the grass family (Poaceae): topological support under different data partitions. PeerJ.

[CR41] Schalamun M, Nagar R, Kainer D, Beavan E, Eccles D, Rathjen JP (2019). Harnessing the MinION: an example of how to establish long-read sequencing in a laboratory using challenging plant tissue from *Eucalyptus pauciflora*. Mol Ecol Resour.

[CR42] Schardl CL, Leuchtmann A, Spiering MJ (2004). Symbioses of grasses with seedborne fungal endophytes. Annu Rev Plant Biol.

[CR43] Schardl CL (2010). The Epichloae, symbionts of the grass subfamily Poöideae. Ann Mo Bot Gard.

[CR44] Schardl CL, Leuchtmann A, Chung KR, Penny D, Siegel MR (1997). Coevolution by common descent of fungal symbionts (*Epichloë* spp.) and grass hosts. Mol Biol Evol.

[CR45] Schardl CL, Craven KD, Speakman S, Stromberg A, Lindstrom A, Yoshida R (2008). A novel test for host-symbiont codivergence indicates ancient origin of fungal endophytes in grasses. Syst Biol.

[CR46] Schardl CL, Young CA, Pan J, Florea S, Takach JE, Panaccione DG (2013). Currencies of mutualisms: sources of alkaloid genes in vertically transmitted epichloae. Toxins (basel).

[CR47] Scott B, Schardl CL (1993). Fungal symbionts of grasses: evolutionary insights and agricultural potential. Trends Microbiol.

[CR48] Scott B, Becker Y, Becker M, Cartwright G, Pérez-Martín J, Di Pietro A (2012). Morphogenesis, growth, and development of the grass symbiont *Epichloë festucae*. Morphogenesis and Pathogenicity in Fungi.

[CR49] Shymanovich T, Charlton ND, Musso AM, Scheerer J, Cech NB, Faeth SH, Young CA (2017). Interspecific and intraspecific hybrid *Epichloë* species symbiotic with the North American native grass *Poa alsodes*. Mycologia.

[CR50] Takach JE, Mittal S, Swoboda GA, Bright SK, Trammell MA, Hopkins AA, Young CA (2012). Genotypic and chemotypic diversity of *Neotyphodium* endophytes in tall fescue from Greece. Appl Environ Microbiol.

[CR51] Tanaka A, Tapper BA, Popay A, Parker EJ, Scott B (2005). A symbiosis expressed non-ribosomal peptide synthetase from a mutualistic fungal endophyte of perennial ryegrass confers protection to the symbiotum from insect herbivory. Mol Microbiol.

[CR52] Tian P, Xu W, Li C, Song H, Wang M, Schardl CL, Nan Z (2020). Phylogenetic relationship and taxonomy of a hybrid *Epichloë* species symbiotic with *Festuca sinensis*. Mycol Progress.

[CR53] Tutin TG, Heywood VH, Burges NA, Moore DM, Valentine DH, Walters SM (2010). Flora Europaea: Alismataceae to orchidaceae (Monocotyledones).

[CR54] Walker BJ, Abeel T, Shea T, Priest M, Abouelliel A, Sakthikumar S (2014). Pilon: an integrated tool for comprehensive microbial variant detection and genome assembly improvement. PLoS ONE.

[CR55] White T, Bruns T, Lee S, Taylor J, Innis M, Gelfand D, Shinsky J, White T (1990). Amplification and direct sequencing of fungal ribosomal RNA genes for phylogenetics. PCR protocols: a guide to methods and applications.

[CR56] White JF, Baldwin NA (1992). A preliminary enumeration of grass endophytes in west central England. Sydowia.

[CR57] Wilson AD, Clement SL, Kaiser WJ, Lester DG (1991). First report of Clavicipitaceous anamorphic endophytes in *Hordeum* species. Plant Dis.

[CR58] Winter DJ, Ganley ARD, Young CA, Liachko I, Schardl CL, Dupont P-Y (2018). Repeat elements organise 3D genome structure and mediate transcription in the filamentous fungus *Epichloë festucae*. PLoS Genet.

[CR59] Young CA, Bryant MK, Christensen MJ, Tapper BA, Bryan GT, Scott B (2005). Molecular cloning and genetic analysis of a symbiosis-expressed gene cluster for lolitrem biosynthesis from a mutualistic endophyte of perennial ryegrass. Mol Genet Genomics.

[CR60] Zacharias D (1993) Flora und Vegetation von Wäldern der Querco-Fagetea im nördlichen Harzvorland Niedersachsens unter besonderer Berücksichtigung der Eichen-Hainbuchen-Mittelwälder [Flora and vegetation of the forests of the Querco-Fagetea in the northern Harz foreland of Lower Saxony with special consideration of the oak-hornbeam middle forests] [Monografie]. Technische Universität Braunschweig, Braunschweig

[CR61] Zhang X, Ren A-Z, Wei Y-K, Lin F, Li C, Liu Z-J, Gao Y-B (2009). Taxonomy, diversity and origins of symbiotic endophytes of *Achnatherum sibiricum* in the Inner Mongolia Steppe of China. FEMS Microbiol Lett.

